# The Impact of Function Focused Care From Acute Care to Home Care and Nursing Homes

**DOI:** 10.1093/geroni/igaa057.2148

**Published:** 2020-12-16

**Authors:** Silke Metzelthin, Sandra Zwakhalen, Barbara Resnick

## Abstract

Functional decline in older adults often lead towards acute or long-term care. In practice, caregivers often focus on completion of care tasks and of prevention of injuries from falls. This task based, safety approach inadvertently results in fewer opportunities for older adults to be actively involved in activities. Further deconditioning and functional decline are common consequences of this inactivity. To prevent or postpone these consequences Function Focused Care (FFC) was developed meaning that caregivers adapt their level of assistance to the capabilities of older adults and stimulate them to do as much as possible by themselves. FFC was first implemented in institutionalized long-term care in the US, but has spread rapidly to other settings (e.g. acute care), target groups (e.g. people with dementia) and countries (e.g. the Netherlands). During this symposium, four presenters from the US and the Netherlands talk about the impact of FFC. The first presentation is about the results of a stepped wedge cluster trial showing a tendency to improve activities of daily living and mobility. The second presentation is about a FFC training program. FFC was feasible to implement in home care and professionals experienced positive changes in knowledge, attitude, skills and support. The next presenter reports about significant improvements regarding time spent in physical activity and a decrease in resistiveness to care in a cluster randomized controlled trial among nursing home residents with dementia. The fourth speaker presents the content and first results of a training program to implement FFC in nursing homes. Nursing Care of Older Adults Interest Group Sponsored Symposium

